# Genome-Wide Identification of *APETALA2/ETHYLENE RESPONSIVE FACTOR* Transcription Factors in *Cucurbita moschata* and Their Involvement in Ethylene Response

**DOI:** 10.3389/fpls.2022.847754

**Published:** 2022-03-15

**Authors:** Qingfei Li, Li Zhang, Peiwen Chen, Chunhui Wu, Huaixia Zhang, Jingping Yuan, Junguo Zhou, Xinzheng Li

**Affiliations:** ^1^College of Horticulture and Landscape, Henan Institute of Science and Technology, Xinxiang, China; ^2^Henan Province Engineering Research Center of Horticultural Plant Resource Utilization and Germplasm Enhancement, Xinxiang, China

**Keywords:** pumpkin (*Cucurbita moschata*), AP2/ERF family, genome-wide identification, ethylene response, transcription factors

## Abstract

*APETALA2/ETHYLENE RESPONSIVE FACTOR* (*AP2/ERF*), a plant-specific transcription factor (TF) family, plays an essential role in the growth and development of plants, and in their response to biotic and abiotic stresses. However, information on *AP2/ERF* in *Cucurbita moschata* (pumpkin), an edible and medicinal vegetable used worldwide, is scarce. A total of 212 *AP2/ERF* genes were identified in the *C. moschata* genome (*CmoAP2/ERFs*). Based on phylogenetic analysis, they were divided into four groups—28 AP2s, 92 ERFs, 86 dehydration-responsive element-binding (DREB) factors, and 6 ABI3/VPs (RAV). The 212 *AP2/ERF* genes were unevenly distributed on the 20 chromosomes of *C. moschata*. The results of structural analysis showed the absence of introns on 132 *CmoAP2/ERFs*. Four pairs of tandem duplication and 155 pairs of segmental duplication events were identified, which indicated that segmental duplications might be the main reason for the expansion of the CmoAP2/ERF family. The analysis of *cis*-regulatory elements (CREs) showed that most of the *CmoAP2/ERFs* contained hormone response elements (ABREs, EREs) in their promoters, suggesting that AP2/ERFs could contribute to the processes regulated by ethylene and abscisic acid. By comparing the transcriptome of ethephon-treated and control plants, we found that 16 *CmoAP2/ERFs* were significantly upregulated after ethephon treatment. Furthermore, we determined the expression patterns of these genes at different developmental stages of female and male flowers. This study provides insights into the identification, classification, physicochemical property, phylogenetic analysis, chromosomal location, gene structure, motif identification, and CRE prediction of the AP2/ERF superfamily in *C. moschata*. Sixteen *CmoAP2/ERF* genes were identified as ethylene-inducible genes. The results of this study will be valuable for understanding the roles of *CmoAP2/ERFs* in ethylene response and should provide a foundation for elucidating the function of AP2/ERF TFs in *C. moschata*.

## Introduction

Plants are exposed to various environmental factors during their growth and development, such as drought, extremes of temperature, salinity, and heavy metals. To combat the various stress, plants have developed complex signaling networks at molecular and cellular levels (Wang et al., 2003; Chinnusamy et al., 2004). Transcription factors (TFs) play important roles in converting stress signals into cellular responses though binding to *cis*-regulatory elements (CREs) of genes involved in stress response (Franco-Zorrilla et al., 2014). Therefore, TFs are important regulators of the growth and development of plants and are involved in their adaptation to environmental factors (Liu et al., 2001; Hir and Bellini, 2013).

The AP2/ERF family of TFs is the largest and unique TF family in plants, the members of which play important roles in regulating diverse processes of plant growth, development, and response to hormone and environmental stress (Riechmann and Meyerowitz, 1998). The AP2/ERF family has been identified in many plants, such as Arabidopsis, rice (Nakano et al., 2006), maize (Zhuang et al., 2010), wheat (Zhuang et al., 2011), radish (Karanja et al., 2019), cucumber (Hu and Liu, 2011), mung bean (Labbo et al., 2018), sugarcane (Li et al., 2020), durum wheat (Faraji et al., 2020), eggplant (Li D. et al., 2021), banana (Lakhwani et al., 2016), and oil palm (Zhou and Yarra, 2021). This family contains at least one highly conserved AP2/ERF DNA binding domain, which consists of 60–70 amino acids. Based on the number of AP2/ERF DNA binding domains and sequence similarities, the AP2/ERF superfamily can be divided into three categories (AP2, EREBP, and RAV). Members of the AP2 family contain two AP2/ERF domains, whereas those with a single AP2/ERF domain are grouped into the EREBP family. The RAV family members consist of two distinct DNA binding domains, the AP2/ERF domain and the B3 domain (Nakano et al., 2006). According to the difference in the 14th and 19th amino acid residues in the AP2/ERF domain, the EREBP family is further divided into ERF and DREB subfamilies. The 14th and 19th amino acids in the ERF subfamily are alanine (A) and aspartic acid (D), respectively, which can specifically bind to a *cis*-acting element, AGCCGCC, of the GCC-box in the promoter region and participate in the regulation of ethylene response and abiotic stress (Ohme-Takagi and Shinshi, 1995). The 14th and 19th amino acids in the DREB subfamily are valine (V) and glutamate (E), respectively, which can specifically bind DRE/CRT elements, and a few members bind GCC-box or CE1 elements. The functions of AP2/ERF family genes have been investigated in several studies. Among them, the members of the AP2 subfamily are involved in the development of floral organs and fruits, as well as in seed germination (Bowman et al., 1989; Elliott et al., 1996). In Arabidopsis, overexpression of soybean *GmAP2* genes led to early flowering and increased the length, width, and area of seeds (Jiang et al., 2020). The EREBP family members mainly respond to abiotic or biotic stress; the DREB subfamily members specifically recognize and bind the dehydration responsive elements (DRE)/C-repeat element (CRT) at the promoter of target genes in response to drought (Dossa et al., 2016), low temperature (Sakuma et al., 2002), and salt stress (Zhao et al., 2018). The ERF family members are involved in ethylene response by binding to GCC-box elements (Ohme-Takagi and Shinshi, 1995), in a stress response-dependent or independent manner. Ectopic expression of *GmERF3*, which binds to the GCC box and DRE/CRT element, in tobacco plants was reported to increase resistance against *Alternaria alternata*, *Ralstonia solanacearum*, and tobacco mosaic virus, and enhanced the tolerance of plants to salinity and dehydration stresses (Zhang et al., 2009). Ectopic expression of *CaERF5* in tobacco led to an upregulation of the expression of defense-related genes and enhanced resistance to *R. solanacearum* infection (Lai et al., 2014). The RAV family proteins are important in signal transduction of plant hormones, such as ethylene and brassinosteroids (Alonso et al., 2003), and participate in the regulation of plant growth and development (Lu et al., 2014; Shin and Nam, 2018; Osnato et al., 2020), and biological and abiotic stress response (Sohn et al., 2006; Li et al., 2011; Fu et al., 2014).

*Cucurbita moschata* is a widely cultivated vegetable crop used both for food and medicine (Lu et al., 2019). As a monoecious plant, sex differentiation of *C. moschata* could be regulated by multiple phytohormones. Among these, ethylene is the main regulator of sex determination in Cucurbitaceae (Manzano et al., 2011). Ethephon is widely used as a ethylene-releasing agent to induce more female flowers to obtain higher yields in Cucurbitaceae crops. As mentioned above, AP2/ERF family members are involved in ethylene response in other crops (Ohme-Takagi and Shinshi, 1995; Alonso et al., 2003). However, the AP2/ERF TF family has not been systematically identified in *C. moschata*, which severely limits the study of the genes in the family.

With the completion of its whole genome sequencing in 2017, the molecular biology research on *C. moschata* has entered a new stage. In this study, the AP2/ERF family members were identified in *C. moschata*. The phylogenetic analysis and characterization of gene structure, conserved motifs, chromosome localization, and CREs were performed. In addition, combined with the transcriptomic data for shoot apices of seedlings treated with ethephon, we analyzed the ethylene response members of the AP2/ERF family in *C. moschata*. The expression patterns of the ethylene-induced genes at different developmental stages of female and male flowers were determined. The results provide a foundation for further exploration of the biological functions of each AP2/ERF family gene and provide clues for unraveling the ethylene response mechanism in *C. moschata*.

## Materials and Methods

### Identification and Classification of AP2/ERF Family Genes in the *Cucurbita moschata* Genome

The *C. moschata* genome sequences^1^ were downloaded to identify the AP2/ERF family members. A Hidden Markov Model search (HMMsearch) was conducted using the HMM profile of the AP2/ERF domain (PF00847), which was obtained using the Pfam program.^2^ The preliminary screening of members was performed using HMMER3.1, with *P* < 0.001. All the searched sequences were then submitted to the SMART tool^3^ and NCBI CDD^4^ to detect the AP2/ERF domain and to remove the sequences without the AP2/ERF domain.

The protein features, including amino acid number, isoelectric point (pI), molecular weight (MW), aliphatic index, grand average of hydropathicity (GRAVY), and instability index, were predicted using ExPASy.^5^

### Chromosomal Distribution, Gene Structure, Conserved Motif, and Phylogenetic Analysis

The information on chromosome distribution of the *AP2/ERF* genes was plotted with TBtools^6^ based on the *C. moschata* genome database. Collinearity analysis within *C. moschata* was performed using the TBtools and MCScanX (Wang et al., 2012; Chen et al., 2020). The values of non-synonymous (Ka) and synonymous (Ks) substitution for each duplicated *AP2/ERF* gene pair were calculated with KaKs_Calculator 2.0 (Wang et al., 2010). The exon–intron organization of the AP2/ERF family genes was displayed using GSDS^7^ (Hu et al., 2015). Conserved motifs were predicted using MEME,^8^ with the parameter of maximum number of motifs being 20. Phylogenetic tree was constructed using the maximum likelihood method and plotted with 1,000 bootstraps using MEGA X (Kumar et al., 2018).

### Analysis of *Cis*-Regulatory Elemens

A sequence 2,000 bp upstream of the transcription start site of each *CmoAP2/ERF* gene was selected to predict CREs, using the PlantCARE tool^9^ (Lescot et al., 2002).

### Ethylene Response Analysis of *CmoAP2/ERF* Genes

The RNA-Seq data of shoot apices subjected to ethephon (100 mg/L) treatment for 4 h, which were previously obtained by our group (NCBI accession number: PRJNA736171), were used to explore the expression patterns of *CmoAP2/ERFs* in response to ethylene. The transcript levels of *CmoAP2/ERFs* were calculated with the fragments per kilobase of exon per million fragments mapped (FPKM) method. The obtained FPKM values of *CmoAP2/ERFs* were Log2 normalized. Heatmap was drawn using the TBtools software for further visualization.

### RNA Extraction and Real-Time PCR Analysis

At the flowering stage of plants, the female and male flower buds with vertical diameters of 0.5, 1.0, and 2.0 cm, which represent different developmental stages, were collected, respectively, and immediately frozen in liquid nitrogen for RNA extraction. RNA was extracted from the samples using the MiniBEST Plant RNA Extraction Kit (Takara, Dalian, China) and then reverse transcribed into cDNA using the PrimeScript™ RT Master Mix (Perfect Real Time) Reagent Kit (Takara, Dalian, China). Real-time PCR was performed on a Bio-Rad IQ5 instrument (Foster City, CA, United States). Based on the results of ethylene response analysis of *CmoAP2/ERF* genes, 16 ethylene-induced genes have been selected to explore their expression patterns at different developmental stages of flower. The primers for *CmoAP2/ERF* genes were designed using the Primer Premier 5.0 software. The sequences of primers are listed in Additional File 1. The reactions were performed as described by the manufacturer. Real-time PCR conditions were as follows: 95 °C for 30 s and 40 cycles of 95 °C for 5 s and 61 °C for 30 s. The RNA expression levels were normalized to that of *ACTIN*. They were calculated using the 2^–ΔΔ*Ct*^ method (Livak and Schmittgen, 2001). The expression of each gene was determined using three biological and three technical replicates.

## Results

### Genome-Wide Identification of *CmoAP2/ERF* Genes

With reference to the *C. moschata* genome database, members of the AP2/ERF family were searched using HMMsearch with a HMM profile of PF00847. After SMART tool and NCBI CDD verification, a total of 212 sequences were identified as candidate *CmoAP2/ERF* genes across the whole genome. The basic features, including amino acid number, aliphatic index, GRAVY, pI, MW, and instability index of the CmoAP2/ERF family genes, are listed in Additional File 2. The identified *CmoAP2/ERF* genes ranged in length from 416 to 22,786 bp. These genes were predicted to encode proteins with 132–1183 amino acids. The MW of CmoAP2/ERF proteins ranged from 14.79 (CmoERF42) to 130.61 (CmoERF36) kDa and the pI varied from 4.36 (CmoERF19) to 11.54 (CmoERF25). There are 73 CmoAP2/ERF proteins with pI > 7 and 139 with pI < 7, which indicates that most of these proteins have a higher proportion of acidic amino acids. The GRAVY score of CmoAP2/ERF proteins ranged from −1.104 (CmoERF68) to −0.018 (CmoDREB12). Most of the *CmoAP2/ERF* genes were predicted to encode unstable proteins with the instability index >40, and only 11 family members, namely CmoRAV2, CmoDREB38, CmoERF34, CmoERF39, CmoERF40, CmoRAV3, CmoDREB65, CmoERF54, CmoRAV5, CmoERF78, and CmoRAV6, were predicted to be stable proteins based on the instability index.

### Phylogenetic Tree Analysis

**FIGURE 1:**
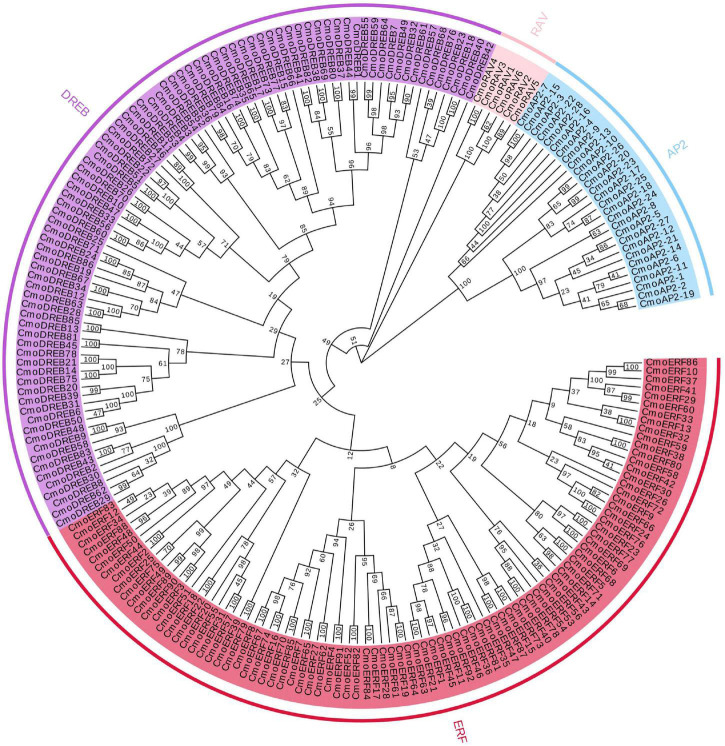
Phylogenetic tree of 212 AP2/ERF family proteins in *Cucurbita moschata*. Red highlighted area shows the ERF group; purple highlighted area shows the DREB group; blue highlighted area shows the AP2 group; pink highlighted area shows the RAV group. The numbers on the branch points represent bootstrap values; scale length represent genetic distance.

To evaluate the evolutionary relationship of the *CmoAP2/ERF* genes, we constructed a phylogenetic tree using the amino acid sequences of proteins encoded by these genes (Figure 1). The *CmoAP2/ERF* genes were grouped into three major classes, EREBP, AP2, and RAV. Twenty-eight genes containing two identical AP2/ERF domains were assigned to the AP2 family, six genes having one AP2/ERF and one B3 domains were assigned to the RAV family, and 178 genes with a single AP2/ERF domain were assigned to the EREBP family. They were further grouped into ERF (92 members, with a conserved Ala and Asp residue located at the 14th and 19th position, respectively) and DREB (86 members, with a conserved Val and Gln residue located at the 14th and 19th position, respectively) subfamilies.

### Gene Structure and Conserved Motif Analysis of the CmoAP2/ERF Family

**FIGURE 2:**
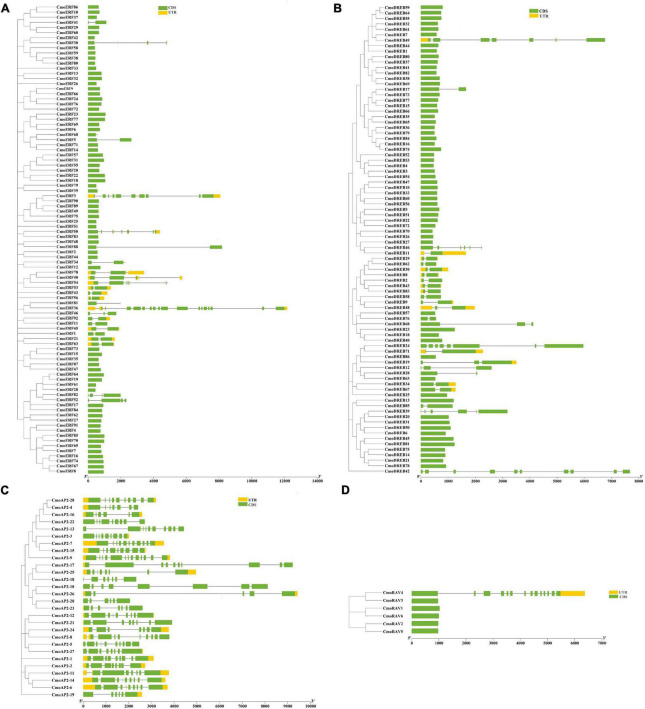
Phylogenetic relationship and gene structure of the 212 CmoAP2/ERF family proteins in *Cucurbita moschata*. CDS (coding sequence) and UTR are represented by the green box and yellow box, respectively, and the black line represents the intron in gene structure analyse. **(A)** ERF subfamily; **(B)** DREB subfamily; **(C)** AP2 subfamily; **(D)** RAV subfamily.

To characterize the gene structure of *CmoAP2/ERF* members, the introns and exons of the *CmoAP2/ERF* genes were examined. As shown in Figure 2A and Additional File 3, in the ERF subfamily, 67 genes have no introns and 25 genes have 1–17 introns. Most of the *DREB* genes have no introns, except for 26 genes, in which the number of introns range from one to nine (Figure 2B). All the *AP2* genes possess 4–12 introns (Figure 2C). Only one *RAV* gene, *CmoRAV4*, has 13 introns, whereas the remaining five *RAV* genes have one intron each (Figure 2D). Overall, 132 members of the CmoAP2/ERF family have no introns and 80 members have 1–17 introns. Among 212 *CmoAP2/ERFs*, 29 have 3′- and 5′-untranslated regions (UTRs); 9 genes lack 5′-UTR, and 10 genes lack 3′-UTR.

**FIGURE 3:**
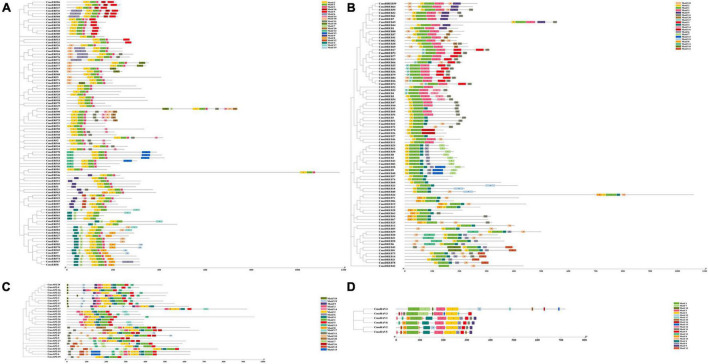
Phylogenetic relationship and conserved motif of the 212 CmoAP2/ERF family proteins in *Cucurbita moschata*. Boxes in different colors represent different conserved motifs. **(A)** ERF subfamily; **(B)** DREB subfamily; **(C)** AP2 subfamily; **(D)** RAV subfamily.

Twenty important conserved motifs in the CmoAP2/ERF proteins were analyzed (Figure 3 and Additional File 4). Motif-1 and -2 are generally present in all the CmoAP2/ERF protein sequences, representing the highly conserved AP2 domain. Besides Motif-1, -2, and -3 are present in most of the ERF protein sequences, except CmoERF64, CmoERF19, CmoERF61, CmoERF28, CmoERF82, CmoERF52, CmoERF17, and CmoERF84. Motif-1 is present in all the DREB protein sequences. Motif-2 is present in most of the DREB protein sequences, except CmoDREB17, CmoDREB73, CmoDREB66, CmoDREB35, CmoDREB65, CmoDREB36, CmoDREB79, CmoDREB84, CmoDREB16, CmoDREB74, CmoDREB53, CmoDREB4, and CmoDREB42. Motif-2, -4, and -6 are present in all the AP2 protein sequences. Motif-5 is commonly found in the AP2 protein, except for CmoAP2-9, whereas interestingly, CmoAP2-9 possess two repeated Motif-18, -19, -9, -3, -14, and -2. Motif-1, -2, and -3 are present in all the RAV protein sequences. The results of conserved motif analysis were generally consistent with the phylogenetic relationship.

### Chromosomal Distribution, Gene Duplication, and Collinearity Analysis of the *CmoAP2/ERF* Genes

**FIGURE 4:**
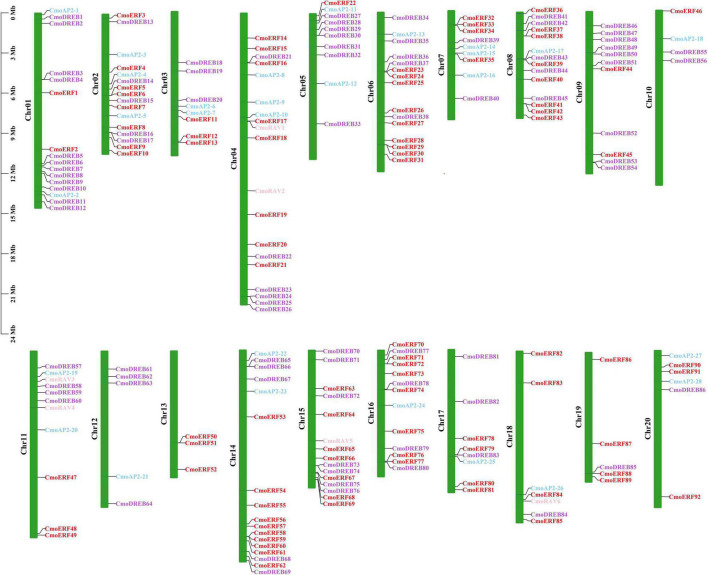
Distribution of *CmoAP2/ERF* genes in *Cucurbita moschata* chromosomes. Different colors distinguish subfamily genes. The number of chromosome was indicated on the left of each chromosome.

To further investigate the gene duplication events in *C. moschata*, the chromosomal distribution of each *CmoAP2/ERF* gene was identified from the genomic data of *C. moschata*. A total of 212 *CmoAP2/ERF* genes were mapped onto 20 chromosomes (Figure 4). Chromosome 04 contained the highest number of *CmoAP2/ERF* genes (19 genes). The lowest number of *CmoAP2/ERF* genes was found on chromosome 13 (3 genes), which were all identified as *ERF* genes. Six *RAV* genes were found to be distributed on four chromosomes, chromosomes 04, 11, 15, and 18. Most of the chromosomes contained a mixture of different family genes, except chromosomes 09, 13, 19, and three chromosomes only contained the EREBP family (ERF and DREB subfamily) genes. The 212 *CmoAP2/ERF* genes were renamed from *CmoERF1* through *CmoERF92*, *CmoDREB1* through *CmoDREB86*, *CmoAP2-1* through *CmoAP2-28*, and *CmoRAV1* through *CmoRAV6*, respectively, based on their order on the chromosome. The precise location of each *CmoAP2/ERF* gene on the chromosomes of *C. moschata* is detailed in Additional File 3.

**FIGURE 5:**
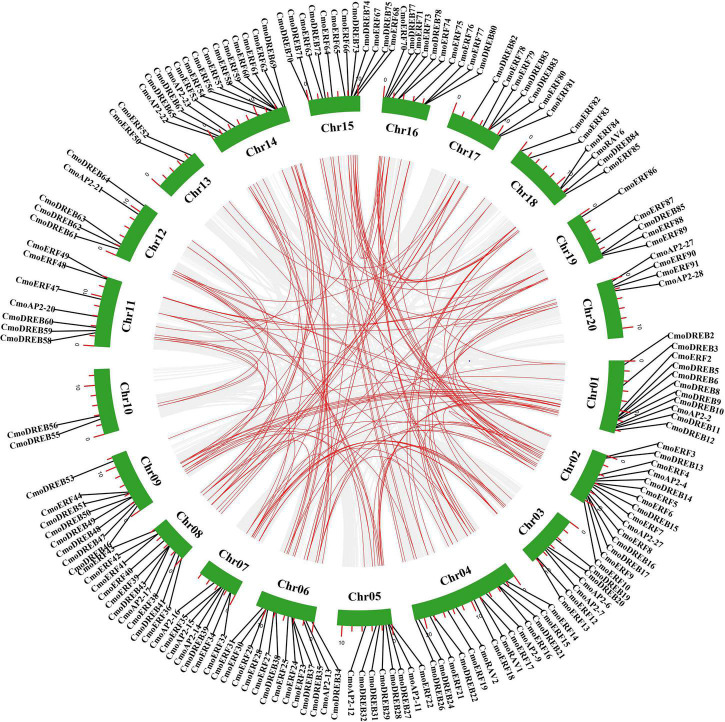
Schematic representations of segmental duplications of *CmoAP2/ERFs*. Gray lines indicate all syntenic blocks in the *Cucurbita moschata* genome, and the red lines indicate duplicated *CmoAP2/ERF* gene pairs. The number of chromosome is shown at the bottom of each chromosome. The red scale bar marked on the chromosome represent the length of the chromosome (Mb).

Gene duplication usually plays a key role in the evolution of gene families. To explore the evolution of *CmoAP2/ERF* genes, we analyzed tandem and segmental duplication events of these genes. Four pairs of tandem duplication genes were detected, of which two pairs were of *ERF* genes, and two were of *DREB* genes (Figure 5 and Additional File 5). A total of 155 pairs of 166 *CmoAP2/ERF* segmental duplication genes was detected, containing *AP2* (21 pairs), *RAV* (2 pairs), *DREB* (53 pairs), and *ERF* (79 pairs) genes (Figure 5 and Additional File 5). These genes accounted for 78.3% of the *CmoAP2/ERF* genes. In addition, Ka/Ks values for *CmoAP2/ERF* genes in tandem and segmental duplications were calculated to determine the selection type that promoted the evolution of the CmoAP2/ERF family. The Ka/Ks values of the four tandem duplication gene pairs varied from 0.12 to 0.21 and those for segmental duplication gene pairs ranged from 0.07 to 0.52, showing that all the gene pairs have a Ka/Ks ratio <1. These results indicate that the evolution of *CmoAP2/ERF* genes is mainly affected under purification selection pressure.

### *Cis*-Regulatory Elemens in the Promoters of CmoAP2/ERF Family Genes

**FIGURE 6:**
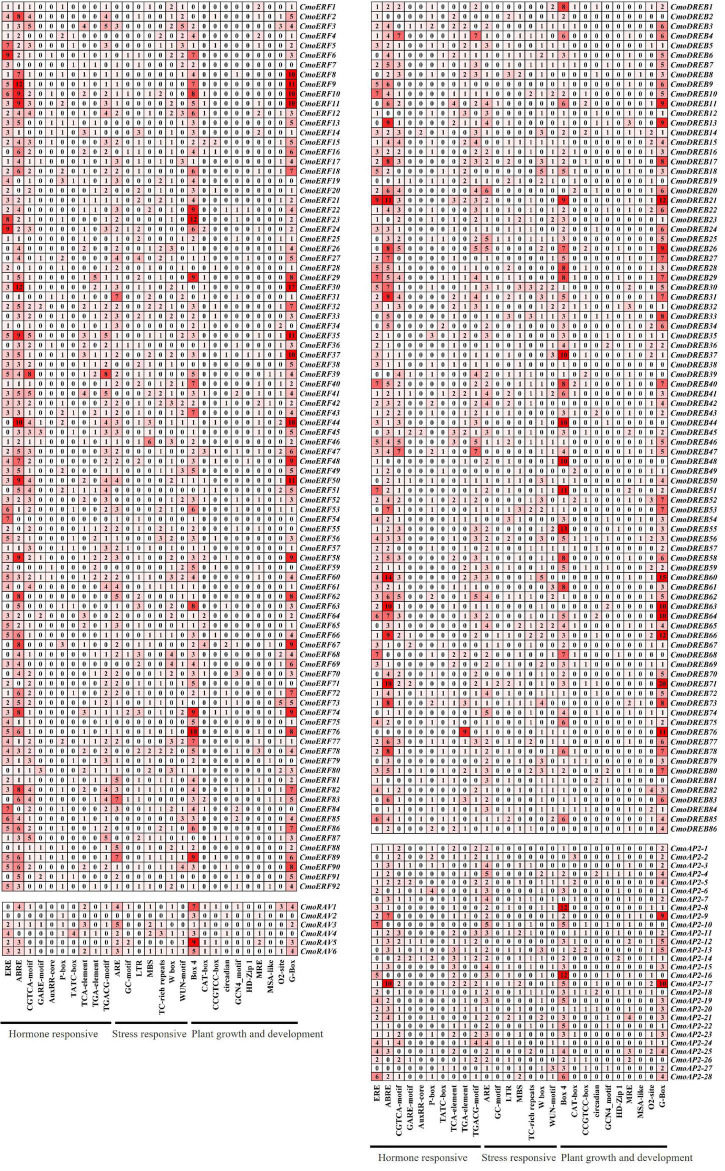
*Cis*-regulatory elements (CREs) in promoter region of *CmoAP2/ERF* genes in *Cucurbita moschata*. The number and the shade of red indicate the number of CREs.

To better understand the regulatory mechanisms of *CmoAP2/ERF* genes, CREs of the promoter regions were studied using PlantCARE. The number of CREs and their functional classification are shown in Figure 6. A total of 5643 elements were predicted in the promoter regions of *CmoAP2/ERF* genes. The results indicated that the CREs mainly included plant hormone responsive elements, stress responsive elements, and plant growth and development elements. Among them, 2458 (43.56%) elements were involved in the response of plants to hormones, such as ethylene (ERE), abscisic acid (ABRE), auxin (TGA-element, AuxRR-core), methyl jasmonate (CGTCA-motif, TGACG-motif), gibberellin (GARE-motif, P-box, TATC-box), and salicylic acid (TCA-element). Among 212 *CmoAP2/ERF* genes, 183 contained abscisic acid responsive elements, ABREs, which accounted for 9.04% of the total number of CREs in the CmoAP2/ERF family. A total of 166 *CmoAP2/ERF* genes contained ethylene responsive elements, EREs, which accounted for 13.34% of the total number of CREs in the CmoAP2/ERF family. The results indicated that most of the *CmoAP2/ERFs* might play a key role in phytohormone regulation, especially for abscisic acid and ethylene. The promoter regions of many *CmoAP2/ERF* genes contain different binding sites involved in stress response. A total of 1121 (19.87%) elements, such as ARE, W box, TC-rich repeats, LTR, MBS, WUN-motif, GC-motif, were involved in stress response. Among 212 *CmoAP2/ERF* genes, 174 contained ARE element, which accounted for 7.02% of the total number of CREs in the CmoAP2/ERF family. A total of 121 *CmoAP2/ERF* genes contained the W box element, which accounted for 3.49% of the total number of CREs in the CmoAP2/ERF family. Overall, 2064 (36.58%) elements, such as G-box, Box 4, O2-site, CAT-box, CCGTCC-box, MRE, circadian, were involved in plant growth and development. G-box and Box 4 were commonly found in the promoter regions of *CmoAP2/ERF* genes. The number of *CmoAP2/ERF* genes that possess G-box and Box 4 elements in the promoter regions was 189 and 188, respectively. This suggests that some members of the CmoAP2/ERF family might play important regulatory roles in the growth and development of plants.

### Expression of *CmoAP2/ERF* Genes in Response to Ethylene Treatment

**FIGURE 7:**
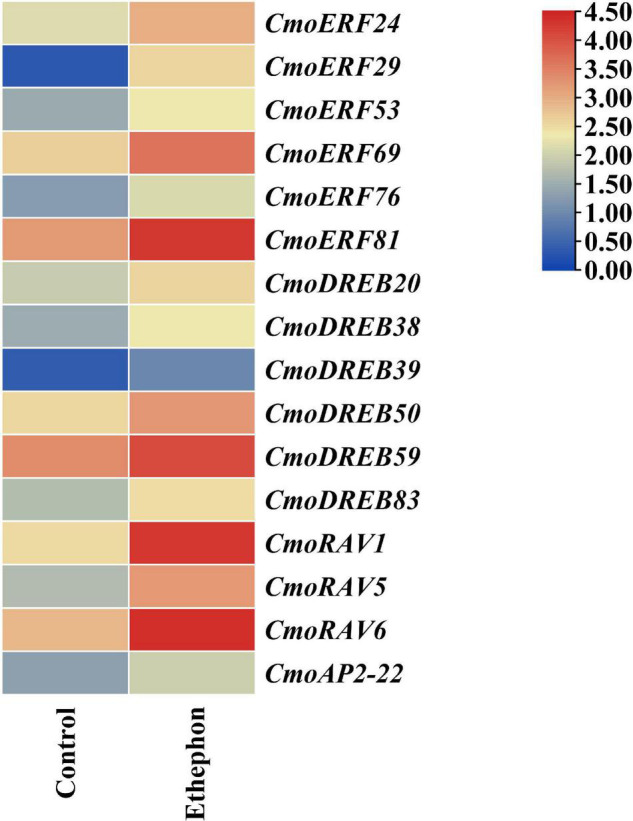
The expression pattern of sixteen CmoAP2/ERF genes in response to ethephon treatment in *Cucurbita moschata*. Expression levels are normalized according to the FPKM and the color scale refers to the deduced FPKM value normalized with Log2.

The results of CRE analysis showed that most of the *CmoAP2/ERF* genes respond to ethylene. To explore the roles of those genes in the response to ethylene, the RNA-Seq data of shoot apical meristem of pumpkin at the seedling stage after ethephon treatment were used to research their expression patterns. The results showed that 16 *CmoAP2/ERF* genes, including *CmoERF24*, *CmoERF29*, *CmoERF53*, *CmoERF69*, *CmoERF76*, *CmoERF81*, *CmoDREB20*, *CmoDREB38*, *CmoDREB39*, *CmoDREB50*, *CmoDREB59*, *CmoDREB83*, *CmoRAV1*, *CmoRAV5*, *CmoRAV6*, and *CmoAP2-22*, were significantly induced (*P*-value < 0.05, fold change ≥1.5). Notably, all these 16 *CmoAP2/ERF* genes were upregulated after ethephon treatment (Figure 7). These 16 ethylene-responsive genes were divided into DREB (6 genes), ERF (6 genes), RAV (3 genes), and AP2 (1 gene) subfamilies. They all have different plant hormone responsive elements. Twelve of the sixteen genes possess EREs, and the remaining four genes have other plant hormone responsive elements.

### Expression Patterns of 16 Ethylene Responsive Genes at Different Developmental Stages of Flower

**FIGURE 8:**
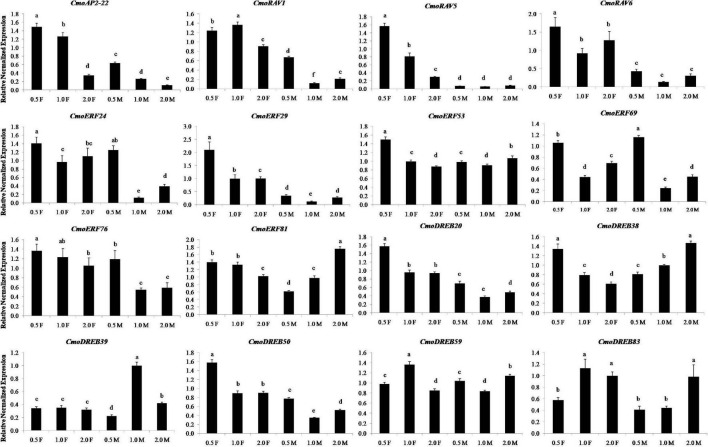
The relative expression levels of the sixteen genes at different developmental stages of flowers in *Cucurbita moschata*. 0.5, 1.0, and 2.0 represent the vertical diameter of flowers and they were measured in centimeters. The F and M represent female flower and male flower, respectively. Relative expression levels are shown as the mean ± SD from three replications. Different letters in a column indicate significant differences between the stages at *p* < 0.05 level.

The results of real-time PCR showed that 16 ethylene-induced genes expressed at different developmental stages of female and male flowers and had different expression patterns (Figure 8). The expression levels of 7 ethylene responsive genes, namely *CmoAP2-22*, *CmoRAV1*, *CmoRAV5*, *CmoRAV6*, *CmoERF29*, *CmoDREB20*, and *CmoDREB50* in the three developmental stages of female flowers were significantly higher than that in male flowers. These genes may be more essential in female flowers than in male flowers. In the three stages of female flowers, the expression levels of 10 ethylene responsive genes, namely *CmoAP2-22*, *CmoRAV5*, *CmoRAV6*, *CmoERF24*, *CmoERF29*, *CmoERF53*, *CmoERF69*, *CmoDREB20*, *CmoDREB38*, *CmoDREB50*, at 0.5 cm stage of female flowers was significantly higher than that in the 1.0 and 2.0 cm stages. Among them, the expression levels of *CmoAP2-22*, *CmoRAV5*, *CmoERF53*, *CmoERF76*, *CmoERF81*, *CmoDREB20*, and *CmoDREB38* showed an obvious downward trend from the 0.5 to 2.0 cm stages of female flowers. In the three stages of male flowers, the expression levels of *CmoAP2-22*, *CmoRAV1*, *CmoERF24*, *CmoERF29*, *CmoERF69*, *CmoERF76*, *CmoDREB20*, and *CmoDREB50* at the 0.5 cm stage of male flowers was significantly higher than that in the 1.0 and 2.0 cm stages. Most of the ethylene responsive genes, except *CmoAP2-22*, *CmoERF81*, *CmoDREB38*, *CmoDREB39*, and *CmoDREB83*, showed the lowest expression in the 1.0 cm stage. This indicates that these genes are expressed in a time-sensitive manner.

## Discussion

As largest plant-specific gene family, the AP2/ERF family plays an important role in regulating plant growth and reproduction, and in coping with environmental stress (Feng et al., 2020). Identification and analysis of gene families provide a basis for systematic study of biological functions of the family members. The information in this regard about the AP2/ERF members of *C. moschata* is limited. In this study, we identified 212 AP2/ERF members in *C. moschata*, benefiting from the publication of the *C. moschata* genome sequence. The number of AP2/ERF family members in *C. moschata* is greater than that in other Cucurbitaceae species, such as cucumber, melon, watermelon, bitter gourd (Lee et al., 2017), which may be due to the complexity of the evolutionary process.

Gene duplication events play key roles in the evolution of a gene family. Tandem duplications usually produce gene clusters or hot regions and segmental duplications generate homologous genes, which combine to bring about the expansion of the gene number (Alonso et al., 2003; Cannon et al., 2004; Zhang and Li, 2018). Four pairs of tandem duplication genes and 155 pairs of segmental duplication events were identified in *C. moschata* chromosomes. The high segmental duplications, which accounted for 78.3% of the *CmoAP2/ERF* genes, may have played important roles in the expansion of the CmoAP2/ERF gene family. In addition, we have found that not all segmentally duplicated genes showed similar functions and stable expressions, which agree with the previous finding (Abdullah et al., 2021). Only three pairs of segmentally duplicated genes (*CmoDREB20-CmoDREB39*, *CmoRAV1*-*CmoRAV6*, *CmoERF24*-*CmoERF76*) showed similar expression that they were up-regulated genes in the treatment of ethephon (Figure 7). 21 pairs of segmentally duplicated genes showed a different expression in the treatment of ethephon, as some genes were found up-regulated while some genes did not express. The expression levels of other segmentally duplicated genes were not affected by ethephon treatment. The paralogous numbers (159) of *AP2/ERF* genes in *C. moschata* was much higher than that in several plant species, including strawberry (14), jujube (18), and pineapple (27). This may be one of the reasons for the large number of *AP2/ERF* genes in *C. moschata*. In addition to tandem duplications and segmental duplications, whole-genome duplication events also affect the number of family members. Studies have found that *C. moschata* may have experienced whole-genome duplication events, core-eudicot common hexaploidization (ECH), cucurbit-common tetraploidization (CCT), cucurbita-specific tetraploidization (CST) (Jiao et al., 2011; Wang et al., 2018; Yu et al., 2021). Therefore, the large gene number of CmoAP2/ERF family can be attributed to tandem duplications, segmental duplications and whole-genome duplication events.

By analyzing the exon–intron structure of the *CmoAP2/ERF* genes, we found that 132 members of the CmoAP2/ERF family, including 67 *ERF* genes, 60 *DREB* genes, and five of the six *RAV* genes, had on introns (Figure 2). Similar results have been found in melon which has been indicated that most ERF family genes have no introns (Ma et al., 2015). It may have relations with the 155 pairs of segmental duplication events in the present study, which further confirmed that segmental duplication results in greater opportunities for losing, rather than acquiring, introns (Lin et al., 2006). These results indicate that *ERF*, *DREB*, and *RAV* gene differentiation might have occurred later in the *C. moschata* evolution. It seems that evolutionary events such as tandem and segmental events in the gene structure as well as in regulatory regions caused a high diversity in functions of *AP2/ERF* genes (Abdullah et al., 2021; Musavizadeh et al., 2021). Conserved motif analyses could provide more information about functional differences among the family members. The results of conserved motifs showed that Motif-1 and -2 are generally present in all the CmoAP2/ERF protein sequences, which indicates that AP2/ERF family genes are highly conserved in the evolution process of different species. Except Motif-1 and -2, the conserved motifs of the CmoAP2/ERF proteins in different subfamilies were different. Motif-2, -4, and -6 were present in all the AP2 protein sequences. Motif-1, -2, and -3 were present in all the RAV protein sequences. These results indicate that the functions of *AP2* and *RAV* gene members may be comparatively conservative. Most of the genes in the same subfamily usually contain similar gene structures and conserved motifs.

Previous study has shown that many *AP2/ERF* genes respond to the plant hormones, such as ethylene and ABA (Xie et al., 2019). It is confirmed by our results of CREs analysis, which indicated that 166 of the 212 *CmoAP2/ERF* genes contained ethylene-responsive elements, EREs, and the ERF subfamily genes contained more number of EREs. The remaining *CmoAP2/ERF* genes possess other hormone response elements (Figure 6). These genes may play key roles in hormone signaling networks. In this study, we found that 16 *CmoAP2/ERF* genes could be positively regulated by ethylene. Among them, 12 genes were of the EREBP family, three were *RAV* genes, and only one gene belonged to the AP2 subfamily. Thus, EREBP family genes may play important roles in the response to ethylene. Similar results have been found in cucumber and melon (Tao et al., 2018). Except for some EREBP family genes, the RAV subfamily members are also involved in ethylene signaling (Alonso et al., 2003) and in the regulation of time of anthesis (Lu et al., 2014). In addition, we found that not all the 16 ethylene-induced genes contain EREs; *CmoRAV1*, *CmoDREB39*, *CmoDREB50*, and *CmoDREB83* contain other plant hormone response elements in their promoter regions. The induction of 16 *CmoAP2/ERF* genes by ethylene might be due to complex plant hormone interactions and network regulatory relationships.

Several studies have shown that *AP2/ERF* genes are involved in flower development. For example, *RAV* genes in rice control the heading date and gynoecium development (Osnato et al., 2020). *CsERF110* has been related to ethylene signaling and floral sex differentiation in cucumber (Tao et al., 2018). An application of exogenous ethylene could induce more female flower in *C. moschata* (Li Q. et al., 2021). The results of qRT-PCR analysis showed that 16 ethylene-induced *CmoAP2/ERF* genes expressed at different developmental stages of female and male flowers. Most of the genes were highly expressed in tender buds (0.5 cm stage). This indicates that these genes may play important roles in flower development. The expression levels of *CmoAP2-22*, *CmoRAV1*, *CmoRAV5*, *CmoRAV6*, *CmoERF29*, *CmoDREB20*, and *CmoDREB50* in female flowers were significantly higher than that in male flowers. Furthermore, these 7 genes were up-regulated during ethylene induction of female flowers. Thus, they may post-ively regulate the formation of female flowers during floral sex differentiation of *C. moschata*. Further researches are required to understand the role of the ethylene-induced genes playing in the process of ethylene induction of female flowers. The results of this study confirm that some of the *AP2/ERF* genes play important roles in flower development.

## Conclusion

In this study, a total of 212 *CmoAP2/ERF* genes were identified from *C. moschata* and their basic biochemical information, phylogenetic relationships, chromosome locations, gene structure, conserved motifs, expression profiles under ethylene treatment were determined. The expression levels of 16 ethylene-induced *CmoAP2/ERF* genes at different developmental stages of female and male flowers were further analyzed. These results should provide an opportunity to understand the roles of *CmoAP2/ERFs* in ethylene responses and in flower development.

## Data Availability Statement

The datasets presented in this study can be found in online repositories. The names of the repository/repositories and accession number(s) can be found in the article/Supplementary Material.

## Author Contributions

QL and LZ conceived the study. QL and PC conducted the molecular experiments. CW, HZ, and JY provided software support. XL and JZ provided plant materials. QL wrote the manuscript. XL revised the manuscript. All authors have read and approved the final manuscript.

## Conflict of Interest

The authors declare that the research was conducted in the absence of any commercial or financial relationships that could be construed as a potential conflict of interest.

## Publisher’s Note

All claims expressed in this article are solely those of the authors and do not necessarily represent those of their affiliated organizations, or those of the publisher, the editors and the reviewers. Any product that may be evaluated in this article, or claim that may be made by its manufacturer, is not guaranteed or endorsed by the publisher.

## Footnotes

1
http://cucurbitgenomics.org/organism/9

2
https://pfam.xfam.org/

3
http://smart.embl.de/

4
https://www.ncbi.nlm.nih.gov/Structure/bwrpsb/bwrpsb.cgi

5
http://web.expasy.org/protparam/

6
https://github.com/CJ-Chen/TBtool

7
http://gsds.gao-lab.org/index.php

8
http://meme-suite.org/tools/meme

9
http://bioinformatics.psb.ugent.be/webtools/plantcare/html/

## Abbreviations

NCBI: national center for biotechnology information
TFs: transcription factors
ERF: ethylene responsive factor
DREB: dehydration-responsive element-binding
CDD: conserved domain database
SMART: simple modular architecture research tool
pI: isoelectric point
MW: molecular weight
GRAVY: grand average hydrophobicity
MEME: multiple EM for motif elicitation
MEGA: molecular evolutionary genetics analysis
Ka/Ks: nonsynonymous substitution rate/synonymous substitution rate
FPKM: fragments per kilobase of transcript per million fragments mapped values
FDR: false discovery rate.
